# Levels of activated platelet-derived microvesicles in patients with soft tissue sarcoma correlate with an increased risk of venous thromboembolism

**DOI:** 10.1186/s12885-017-3515-y

**Published:** 2017-08-07

**Authors:** A. Fricke, P. V. Ullrich, A. F. V. Cimniak, C. Becherer, M. Follo, J. Heinz, J. Scholber, G. W. Herget, O. Hauschild, U. A. Wittel, G. B. Stark, H. Bannasch, D. Braig, S. U. Eisenhardt

**Affiliations:** 1 Department of Plastic and Hand Surgery, Medical Center - Faculty of Medicine, University of Freiburg, Hugstetter Straße 55, 79106 Freiburg, Germany; 2 Department of Hematology, Oncology and Stem Cell Transplantation, Medical Center - Faculty of Medicine, University of Freiburg, Hugstetter Straße 55, 79106 Freiburg, Germany; 3 Department of Radiation Oncology, Medical Center - Faculty of Medicine, University of Freiburg, Robert-Koch-Straße 3, 79106 Freiburg, Germany; 4 Department of Orthopedics and Traumatology, Medical Center - Faculty of Medicine, University of Freiburg, Hugstetter Straße 55, 79106 Freiburg, Germany; 5 Department of General and Visceral Surgery, Medical Center - Faculty of Medicine, University of Freiburg, Hugstetter Straße 55, 79106 Freiburg, Germany

**Keywords:** Microvesicles, Sarcoma, biomarker, FACS

## Abstract

**Background:**

Microvesicles are small vesicles expressing specific antigens from their cells of origin. Elevated levels of microvesicles have been shown to be associated with coagulation disorders as well as with different types of malignancies. This study aims to evaluate a possible correlation of different microvesicle subpopulations with a positive history of venous thromboembolism (VTE) in patients with soft tissue sarcoma.

**Methods:**

Annexin V - positive microvesicles, leukocyte (CD45-positive), platelet (CD61-positive), activated platelet (CD62P-, CD63-positive), endothelium-derived (CD62E-positive) and tissue-factor (CD142-positive) microvesicles were identified in the peripheral blood of patients with soft tissue sarcoma (*n* = 39) and healthy controls (*n* = 17) using fluorescence-activated cell sorting (FACS).

**Results:**

Both the total amount of Annexin V-positive microvesicles and levels of endothelium-derived (CD62E-positive) microvesicles were shown to decrease significantly after tumor resection (*n* = 18, *p* = 0.0395 and *p* = 0.0109, respectively). Furthermore, the total amount of Annexin V – positive microvesicles as well as leukocyte (CD45-positive) and endothelium-derived (CD62E-positive) microvesicles were significantly higher in patients with grade 3 (G3) soft tissue sarcoma (*n* = 9) compared to healthy controls (*n* = 17) (*p* = 0.0304, *p* = 0.0254 and *p* = 0.0357, respectively). Moreover, patients with G3 soft tissue sarcoma (*n* = 9) presented higher levels of Annexin V-positive and endothelium-derived (CD62E-positive) microvesicles compared to patients with grade 2 (G2) soft tissue sarcoma (*n* = 8) (*p* = 0.0483 and *p* = 0.0045). Patients with grade 1 (G1) soft tissue sarcoma (*n* = 3) presented with significantly lower levels of platelet (CD61-positive) microvesicles than patients with G3 soft tissue sarcoma (*n* = 9) (*p* = 0.0150).

In patients with a positive history of VTE (*n* = 11), significantly higher levels of activated platelet (CD62P- and CD63-positive) microvesicles (*p* = 0.0078 and *p* = 0.0450, respectively) were found compared to patients without a history of VTE (*n* = 28).

**Conclusion:**

We found significantly higher levels of Annexin V-positive and endothelium-derived (CD62E-positive) microvesicles to be circulating in the peripheral blood of patients with G3 soft tissue sarcoma compared to patients with G2 soft tissue sarcoma. Furthermore, we showed that high counts of activated platelet-derived microvesicles correlate with the occurrence of VTE. Thus, the detection of these microvesicles might be an interesting new tool for early diagnosis of soft tissue sarcoma patients with increased risk for VTE, possibly facilitating VTE prevention by earlier use of thromboprophylaxis.

**Electronic supplementary material:**

The online version of this article (doi:10.1186/s12885-017-3515-y) contains supplementary material, which is available to authorized users.

## Background

Soft tissue sarcoma are a heterogeneous group of malignant tumors of mesenchymal origin, accounting for approximately 1% of all malignancies in adults. Microvesicles are small vesicles expressing specific antigens from their cells of origin [1]. Elevated levels of microvesicles have been shown to be associated with inflammatory, cardiovascular and autoimmune disorders as well as with different types of malignancies [2–7]. Moreover, platelet microvesicles, which constitute approximately two thirds of circulating microvesicles in human peripheral blood [8], have been found to play an important role in angiogenesis and the development of metastasis in different malignancies [9, 10], as well as provoking an immune response in hematopoietic, endothelial and monocytic cells through the induction of differential gene expression [11, 12]. Furthermore, it has been proven that circulating microvesicles can transfer tissue factor (TF) [13] or bioactive lipids such as arachidonic acid to platelets and endothelial cells, activating platelets and thus being of importance in the initiation of coagulation [14]. It has also been found that the level of circulating platelet microvesicles correlates with the risk of venous thromboembolism (VTE) in cancer patients [15]. The thrombogenicity of circulating microvesicles has been shown to be mostly due to negatively charged phospholipids such as phosphatidylserine, as well as to the presence of TF, a transmembrane receptor which plays an important role in the initiation of coagulation, in the microvesicle membrane [16]. Interestingly, Davila et al. found that tumor cells release TF – positive microvesicles into the circulation [17], which are associated with VTE in different malignancies [18]. Thus, the higher risk for VTE in cancer patients [19–21], which is elevated in patients with soft tissue sarcoma [22], might be due to circulating microvesicles of different cellular origin. Toth et al. found that the levels of platelet (CD61-positive), activated platelet (platelets which have undergone degranulation and are CD62P- and CD63-positive), endothelium-derived (CD62E-positive), leukocyte (CD45-positive) and TF-bearing (CD142-positive) microvesicles as well as the total amount of Annexin V - positive microvesicles correlate with tumor size in breast cancer patients [15]. Thus, the aim of our study was to analyze the number of these microvesicle subpopulations in patients with soft tissue sarcoma before and after tumor resection as well as to examine a possible correlation of the microvesicle subpopulations with tumor grading and a positive history of VTE.

## Methods

### Study population

All patients taking part in the study were treated by specialists in the Comprehensive Cancer Center Freiburg (CCCF). Patients with a history of cancer other than sarcoma, any type of systemic inflammatory disease, autoimmune or coagulation disorder were excluded. Furthermore, patients undergoing neoadjuvant chemotherapy or radiotherapy prior to blood withdrawal were excluded from the study. Due to these strict criteria, 39 patients out of 94 soft tissue sarcoma patients treated from 31.01.2014 until 31.01.2016 were included in the study. Diagnosis of the different sarcoma subtypes was confirmed by two independent pathologists.

Patients with localized disease (*n* = 20) were divided into three groups according to their tumor’s histologic grading. The category of grade 1 (G1) soft tissue sarcoma (*n* = 3) included one dermatofibrosarcoma protuberans (DFSP) and two liposarcoma. The group of patients with grade 2 (G2) soft tissue sarcoma (*n* = 8) consisted of one extraskeletal myxoid chondrosarcoma, two leiomyosarcoma, three liposarcoma, one myxoid malignant fibrous histiocytoma and one synovial sarcoma. The group of patients with grade 3 (G3) soft tissue sarcoma (*n* = 9) was composed of two leiomyosarcoma, one liposarcoma, one myofibroblastic sarcoma, one myxoid fibrosarcoma and four pleomorphic sarcoma.

The group of patients with metastasized soft tissue sarcoma (M1) (*n* = 19) consisted of one intimal sarcoma of the pulmonary artery, six leiomyosarcoma, two liposarcoma, one malignant peripheral nerve sheath tumor (MPNST), two pleomorphic sarcoma, one spindle-cell sarcoma and six synovial sarcoma.

Furthermore, all groups were analyzed with regard to their history of VTE, including past events of deep vein thrombosis, pulmonary embolism or other venous thrombotic events. Within the G2 group (*n* = 8), three patients had a positive history of VTE, while in the G1 (*n* = 3) and G3 groups (*n* = 9), no history of VTE was detected. In the group of patients with metastasized disease (*n* = 19), eight patients had a positive history of VTE. The control group (*n* = 17) included healthy adults without any type of systemic inflammatory disease, autoimmune or coagulation disorder. Mean age of the healthy controls was 48.0 years; while mean body mass index (BMI) was 24.2 kg/m^2^. All patients and healthy controls were of European origin.

### Blood sampling

All blood samples were collected by puncture of the antecubital vein without tourniquet through a 21-gauge needle. The first 3 ml of blood were discarded. Each 9 ml of blood was collected in heparin. When patients presented with localized disease, blood was withdrawn within 1 day before surgery and 12–15 days after surgery.

### Preparation of samples

Blood samples were double centrifuged at 2500 *g* for 15 min at room temperature (RT) to obtain cell-free plasma; then they were snap-frozen in liquid nitrogen and stored at −80 °C until further procedures were carried out.

### Flow cytometry

Annexin V – positive leukocyte (CD45/leukocyte common antigen-positive), platelet (CD61/Integrin β3-positive), activated platelet (CD62P/P-selectin-, CD63/gp55-positive) [7], endothelium-derived (CD62E/E-selectin-positive), and tissue-factor-bearing (CD142-positive) microvesicles were identified in the cell-free plasma of patients with soft tissue sarcoma and healthy controls using fluorescence-activated cell sorting (FACS). Annexin V conjugated with fluorescein isothiocynate (FITC), mouse anti-human CD45, CD61, CD62P, CD63, CD62E and CD142 conjugated with Phycoerythrin (PE) and TruCOUNT™ beads were purchased from BD Biosciences. Each 10 μl of cell-free plasma was incubated with 90 μl of Annexin-Binding Buffer (25 mM CaCl_2,_ 100 mM HEPES, 1.4 M NaCl; pH 7.4); 1 μl Annexin V and 2 μl cell-specific antibody for 20 min. Annexin-Binding Buffer was filtered twice through a 0.22 μm filter before use. For calculation of total counts, TruCOUNT™ beads were added immediately prior to analysis by flow cytometry at a final concentration of ten beads per μl total volume. Gain settings were adjusted to place the TruCOUNT™ beads in the upper decade for scatter as described by Jayachandran et al. [23]. Microvesicles are defined as being smaller than 1 μm, so we used 1 μm calibrating beads (Latexbeads, amine-modified polystyrene, fluorescent yellow-green; Sigma, St. Louis, MO, USA) and log scaling in both the forward scatter and side scatter parameters to help define the microvesicle gate. We then selected for vesicles which were positive for Annexin V in combination with the different cell-specific markers which were used. The gating boundaries were set with the help of isotype control fluorescent antibodies (Right Reference Standard Phycoerythrin/Fluorescein; Bangs Laboratories, Fishers, IN, USA).

All analyses were performed using a LSR Fortessa Cell Analyzer (BD Biosciences) with 488 nm excitation used for FITC and 561 nm excitation used for PE. Microvesicle counts were calculated from the nominal number of beads added per volume of sample, with 500 TruCOUNT™ bead events per analysis as described by Jayachandran et al. [23]. For data analyses, the FlowJo Software, Version 10 (FlowJo, Ashland, OR, USA) was used. Representative fluorescence-activated cell sorting (FACS) dot plots are shown in Additional file 1.

### Statistics

Microvesicle counts and hemoglobin (Hb), platelet and leukocyte counts of pre- and post-operative patient groups were compared using the paired Student’s *t*-test; the remaining groups were compared using Student’s *t*-test for independent samples. *p*-values were rounded to 4 significant digits; *p*-values below 0.05 were considered statistically significant.

## Results

**Fig. 1:**
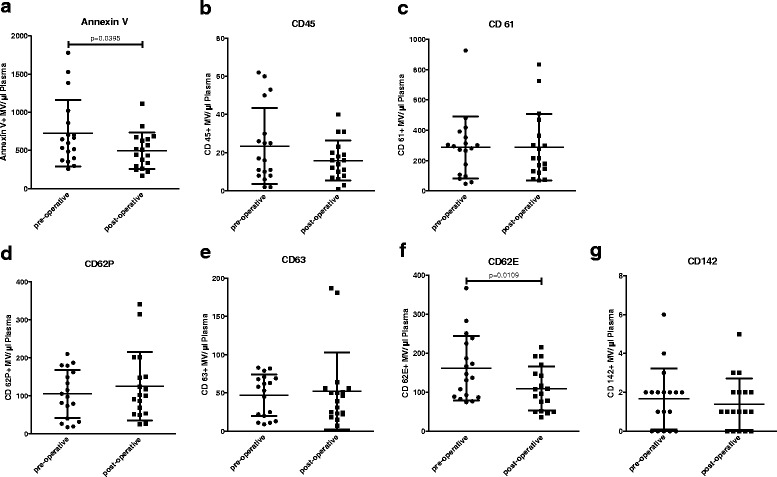
Pre- and post-operative microvesicle counts of patients with localized soft tissue sarcoma who underwent R0-tumor resection. Data are presented as mean value ± standard deviation (SD). *p*-values were determined using the paired Student’s *t*-tests. **a**. Annexin V = total amount of microvesicles as detected using FACS. **b**. CD45 (leukocyte common antigen) = leukocyte-derived microvesicles. **c**. CD61 (Integrin β3) = platelet-derived microvesicles. **d**. CD62P (P-selectin) = activated platelet-derived microvesicles. **e**. CD63 (gp55) = activated platelet-derived microvesicles. **f**. CD62E (E-selectin) = endothelium-derived microvesicles. **g**. CD142 (Tissue Factor) = microvesicles carrying tissue factor

The total amount of Annexin V positive microvesicles and levels of endothelium-derived (CD62E-positive) microvesicles in the peripheral blood of patients with soft tissue sarcoma were shown to decrease significantly after tumor resection (*n* = 18, *p* = 0.0395 and *p* = 0.0109, respectively; Fig. 1).

Pre- and post-operative Hb and platelet counts of patients with localized soft tissue sarcoma undergoing resection differed significantly (*p* = 0.0037 and *p* = 0.0054, respectively) (Table 1), with decreased Hb and increased platelet counts after tumor resection.

**Table 1:** Demographic patient data and blood count of patients who underwent tumor resection

	Pre-operative (*n* = 18)	Post-operative (*n* = 18)	*p*-value pre- vs. post-operative
Age (years)	57.2 ± 13.0		
BMI (kg/m^2^)	25.2 ± 3.1		
Hb (g/dl)	13.5 ± 1.5	11.3 ± 2.4	**0.0037**
Platelets (th/μl)	255.0 ± 58.0	387.0 ± 166.0	**0.0054**
Leukocytes (th/μl)	6.9 ± 1.5	7.51 ± 2.0	0.3122

Demographic patient data (age, BMI) and pre- and postoperative blood counts (hemoglobin (Hb), platelet and leukocyte counts) of patients with localized soft tissue sarcoma undergoing tumor resection. Data are presented as mean value ± standard deviation (SD). *p*-values were determined using paired Student’s *t*-tests. *p*-values <0.05 are marked in bold

**Fig. 2:**
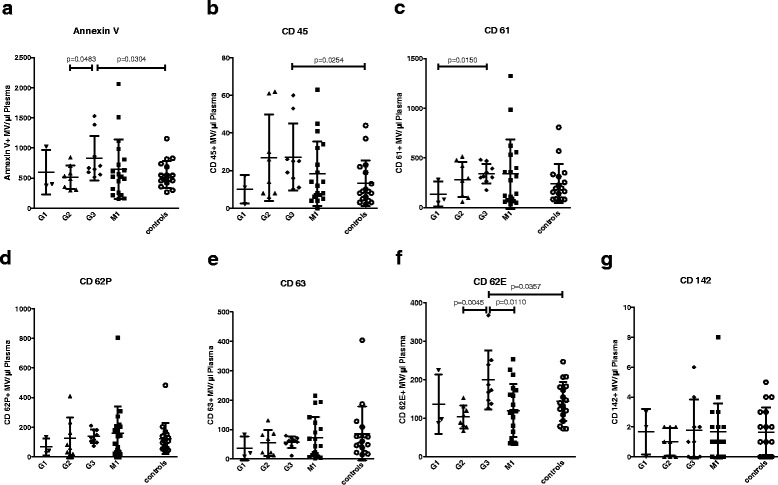
Microvesicle counts of healthy controls, patients with localized soft tissue sarcoma (G1, G2 and G3) and patients with metastasized soft tissue sarcoma (M1). Data are presented as mean value ± standard deviation (SD). *p*-values were determined using Student’s *t*-tests for independent samples. **a**. Annexin V = total amount of microvesicles as detected using FACS. **b**. CD45 (leukocyte common antigen) = leukocyte-derived microvesicles. **c**. CD61 (Integrin β3) = platelet-derived microvesicles. **d**. CD62P (P-selectin) = activated platelet-derived microvesicles. **e**. CD63 (gp55) = activated platelet-derived microvesicles. **f**. CD62E (E-selectin) = endothelium-derived microvesicles. **g**. CD142 (Tissue Factor) = microvesicles carrying tissue factor

The total amount of Annexin V-positive microvesicles as well as leukocyte (CD45-positive) and endothelium-derived (CD62E-positive) microvesicles were significantly higher in patients with G3 soft tissue sarcoma (*n* = 9) compared to healthy controls (*n* = 17) (*p* = 0.0304, *p* = 0.0254 and *p* = 0.0357, respectively; Fig. 2).

Moreover, patients with G3 soft tissue sarcoma (*n* = 9) presented with higher levels of Annexin V-positive and endothelium-derived (CD62E-positive) microvesicles compared to patients with G2 soft tissue sarcoma (*n* = 8) (*p* = 0.0483 and *p* = 0.0045). Patients with G1 soft tissue sarcoma (*n* = 3) showed significantly lower levels of platelet-derived (CD61-positive) microvesicles than patients with G3 soft tissue sarcoma (*n* = 9) (*p* = 0.0150; Fig. 2). However, when comparing all pre-operative samples including G1, G2 and G3 sarcoma patients with and without history of VTE to patients with metastasized disease and healthy donors, no statistically significant differences were detected. Furthermore, we did not find statistically significant differences of microvesicle counts when comparing different sarcoma subtypes.

When comparing Hb, platelet and leukocyte counts of patients with G3 soft tissue sarcoma to healthy controls, Hb values of the healthy controls were significantly higher than Hb values of patients with G3 localized soft tissue sarcoma (*p* = 0.0285). Furthermore, patients with G3 soft tissue sarcoma exhibited significantly higher platelet counts compared to healthy controls (*p* = 0.0461), as well as significantly higher leukocyte counts (*p* = 0.0299). There were no significant differences concerning Hb, platelet or leukocyte counts when comparing patients with G3 soft tissue sarcoma to patients with G1 or G2 soft tissue sarcoma.

When comparing Hb, platelet and leukocyte counts of patients with metastasized soft tissue sarcoma to healthy controls, Hb values of the healthy controls were significantly higher than Hb values of patients with metastasized soft tissue sarcoma (*p* < 0.0001) (Table 2).

**Table 2:** Demographic patient data and blood count of healthy donors and patients with localized disease and metastasized disease

	Healthy controls (*n* = 17)	G1 (localized disease) (*n* = 3)	G2 (localized disease) (*n* = 8)	G3 (localized disease) (*n* = 9)	Metastasized disease (M1) (*n* = 19)	*p*-value G1 vs G3 (localized disease)	*p*-value G2 vs G3 (localized disease)	*p*-value controls vs G2 (localized disease)	*p*-value controls vs G3 (localized disease)	*p*-value controls vs M1 (metastasized disease)
Age (years)	48.0 ± 10.0	48.7 ± 6.9	63.7 ± 11.4	56.7 ± 13.7	54.8 ± 12.4	0.3904	0.1190	**0.0027**	0.0875	0.0873
BMI (kg/m^2^)	24.2 ± 3.4	22.6 ± 3.3	24.7 ± 3.0	25.4 ± 2.6	24.4 ± 4.4	0.2872	0.9434	0.7498	0.4440	0.9049
Hb (g/dl)	14.6 ± 1.0	14.1 ± 1.0	12.4 ± 2.4	13.3 ± 1.7	12.0 ± 1.8	0.5022	0.7927	**0.0041**	**0.0285**	**< 0.0001**
Platelets (th/μl)	227.3 ± 45.5	225.3 ± 46.2	310.4 ± 116.4	289.9 ± 93.7	293.2 ± 140.8	0.3423	0.4906	**0.0222**	**0.0461**	0.0828
Leukocytes (th/μl)	5.8 ± 1.2	6.9 ± 0.8	7.5 ± 2.7	7.3 ± 2.0	7.85 ± 5.5	0.7512	0.3619	**0.0401**	**0.0299**	0.1524

Demographic patient data (age, BMI) and blood counts (hemoglobin (Hb), platelet and leukocyte counts) of patients with localized disease (G1, G2 and G3) and metastasized disease (M1). Data are presented as mean value ± standard deviation (SD). *p*-values were determined using Student’s *t*-tests for independent samples. *p*-values <0.05 are marked in bold

**Fig. 3:**
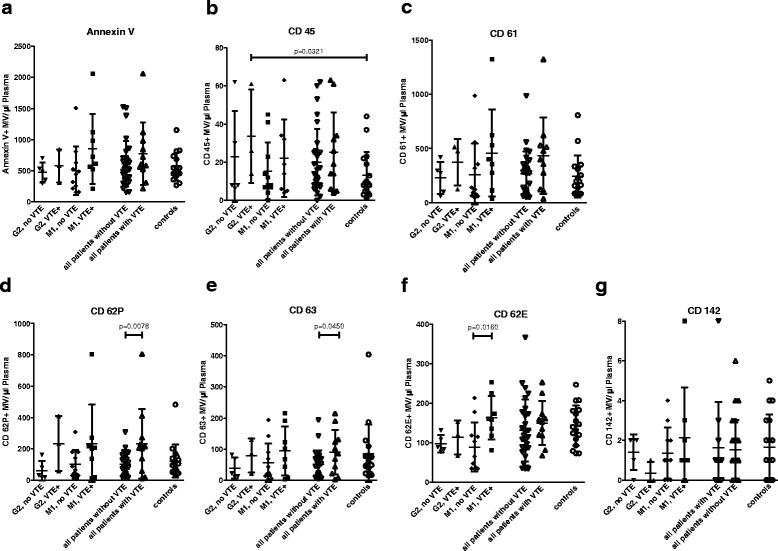
Microvesicle counts of patients with soft tissue with and without a history of venous thromboembolism (VTE). Comparison of G2 soft tissue sarcoma patients with and without positive history of VTE, M1 soft tissue sarcoma patients with and without positive history of VTE and all soft tissue sarcoma patients with and without a positive history of VTE. G1 and G3 soft tissue sarcoma patients did not exhibit a positive history of VTE. Data are presented as mean value ± standard deviation (SD). *p*-values were determined using Student’s *t*-tests for independent samples. **a**. Annexin V = total amount of microvesicles as detected using FACS. **b**. CD45 (leukocyte common antigen) = leukocyte-derived microvesicles. **c**. CD61 (Integrin β3) = platelet-derived microvesicles. **d**. CD62P (P-selectin) = activated platelet-derived microvesicles. **e**. CD63 (gp55) = activated platelet-derived microvesicles. **f**. CD62E (E-selectin) = endothelium-derived microvesicles. **g**. CD142 (Tissue Factor) = microvesicles carrying tissue factor

A positive history of VTE was present in 42.1% of patients with metastases and 15.0% of patients with localized disease. Comparing all soft tissue sarcoma patients with a positive history of VTE (*n* = 11) to all soft tissue sarcoma patients without a history of VTE (*n* = 28), we found significantly higher levels of activated platelet-derived (CD62P- and CD63-positive) microvesicles (*p* = 0.0078 and *p* = 0.0450, respectively; Fig. 3). Patients with localized G2 soft tissue sarcoma with a positive history of VTE (*n* = 3) showed significantly higher values of leukocyte (CD45-positive) microvesicles compared to healthy donors (*p* = 0.0321). Within the group of metastasized soft tissue sarcoma, patients with a positive history of VTE (*n* = 8) presented with significantly higher levels of endothelium-derived (CD62E-positive) microvesicles than the patients without VTE (*p* = 0.0160).

Comparing Hb, platelet and leukocyte counts, patients with localized G2 soft tissue sarcoma with a positive history of VTE (*n* = 3) exhibited significantly lower Hb-values whereas the platelet and leukocyte counts were significantly higher compared to healthy controls (*p* = 0.0003, *p* = 0.0018 and *p* = 0.0032, respectively; Table 3). Within the groups of patients with metastasized soft tissue sarcoma with or without a history of VTE, Hb, platelet and leukocyte counts did not differ significantly. However, soft tissue sarcoma patients with a history of VTE (*n* = 11) presented with significantly higher platelet counts (*p* = 0.0186) than patients without a history of VTE (*n* = 28; Table 3).

**Table 3:** Demographic patient data (age, BMI) and blood count (hemoglobin (Hb), platelet and leukocyte counts) of soft tissue sarcoma patients with and without a history of venous thromboembolism (VTE)

	G2 without a history of VTE (*n* = 5)	G2 with history of VTE (*n* = 3)	M1 without a history of VTE (*n* = 11)	M1 with history of VTE (*n* = 8)	All sarcoma patients without history of VTE (*n* = 28)	All sarcoma patients with history of VTE (*n* = 11)	Healthy controls (*n* = 17)	*p*-value of G2 patients with VTE vs controls	*p*-value of M1 patients with vs without VTE	*p*-value of all patients with VTE vs without VTE
Age (years)	69.4 ± 10.7	54.3 ± 3.9	56.8 ± 10.3	52.13 ± 14.4	58.1 ± 12.6	52.7 ± 12.4	48 ± 10.0	0.3179	0.4426	0.2460
BMI (kg/m^2^)	25.5 ± 2.5	23.3 ± 3.2	24.4 ± 4.2	24.3 ± 4.8	24.7 ± 3.6	24.1 ± 4.4	24.2 ± 3.4	0.7287	0.9756	0.6577
Hb (g/dl)	13.5 ± 0.9	10.5 ± 2.7	11.6 ± 1.6	12.7 ± 1.8	12.8 ± 1.8	12 ± 2.4	14,63 ± 1.0	**0.0003**	0.2561	0.3167
Platelets (th/μl)	256.0 ± 16.5	401.0 ± 150.1	254.0 ± 123.3	354.9 ± 144.4	261.8 ± 98.3	368.7 ± 147.7	227.3 ± 45.5	**0.0018**	0.1554	**0.0186**
Leukocytes (th/μl)	6.3 ± 1.0	9.5 ± 3.3	7.8 ± 6.5	7.9 ± 3.6	7.3 ± 4.3	8.4 ± 3.6	5.8 ± 1.2	**0.0032**	0.9773	0.4848

Data are presented as mean value ± standard deviation (SD). *p*-values were determined using Student’s *t*-tests for independent samples. *p*-values <0.05 are marked in bold

## Discussion

In our study, we showed that levels of Annexin V-positive and endothelium-derived (CD62E-positive) microvesicles decrease significantly after tumor resection (Fig. 1). Although increased platelet counts were found after tumor resection (Table 1), levels of platelet-derived (CD61-positive) microvesicles and microvesicles derived from activated platelets (CD62P- and CD63-positive) were not significantly altered after tumor resection (Fig. 1), which demonstrates that our findings were not due to blood count alterations.

We furthermore found that the total amounts of Annexin V-positive microvesicles as well as leukocyte (CD45-positive) and endothelium-derived (CD62E-positive) microvesicles were significantly higher in patients with G3 soft tissue sarcoma compared to healthy controls. Moreover, patients with G3 soft tissue sarcoma exhibited significantly higher levels of Annexin V-positive and endothelium-derived (CD62E-positive) microvesicles compared to patients with G2 soft tissue sarcoma. In contrast to these results, a study by Toth et al. found that although leukocyte-derived microvesicle levels differed significantly between breast cancer patients and controls, no differences in endothelium-derived microvesicle levels were detected [24]. Thus, an elevation in endothelium-derived microvesicle counts does not seem to be common among cancer patients.

The fact that patients with metastasized disease did not show significantly higher microvesicle counts than healthy controls or patients with localized disease might partly be due to the fact that there is a high variation in microvesicle counts within different patients, which is in agreement with other studies analyzing microvesicle counts in cancer patients [15, 24–26]. Furthermore, pre-operative samples of patients with localized soft tissue sarcoma were composed of G1, G2 and G3 sarcoma samples, including patients with and without history of VTE; which might have been a further reason for the fact that no statistically significant differences in microvesicle counts were detected when comparing patients with metastasized disease to pre-operative samples of patients with localized disease or healthy donors.

Of note, the patients’ blood was collected in heparin since it has been demonstrated that microvesicle counts collected in calcium-chelating anticoagulants such as citrate or EDTA lead to a loss of microvesicles [23]. Furthermore, we chose not to use ultracentrifugation of microvesicles prior to analysis by FACS, since microvesicles might be lost when resuspending the microvesicle pellet; moreover, we consider this method to potentially lead to clotting of microvesicles. Thus, blood samples were double centrifuged at 2500 *g* for 15 min at room temperature (RT) to obtain cell-free plasma prior to FACS analysis as previously described by Laresche et al. [27].

A positive history of VTE was present in 42.1% of patients with metastases and 15.0% of patients with localized disease. Thus, we found higher VTE rates to occur in an adult population when compared to a study conducted by Paz-Priel et al., who demonstrated that VTE developed in 23% of pediatric sarcoma patients with metastases versus 10% with localized disease [22].

Patients with localized (G2) soft tissue sarcoma with a positive history of VTE presented with significantly higher values of leukocyte (CD45-positive) microvesicles compared to healthy donors, which could be explained by the significantly higher leukocyte counts in patients with localized G2 soft tissue sarcoma with VTE (*p* = 0.0032). Within the group of metastasized soft tissue sarcoma, patients with a positive history of VTE showed significantly higher levels of endothelium-derived (CD62E-positive) microvesicles than the patients without VTE, while Hb, platelet and leukocyte counts did not differ significantly between these groups.

Within the G2 group (*n* = 8) three patients had a positive history of VTE, while in the G1 (*n* = 3) and G3 groups (*n* = 9), no history of VTE was shown. In the group of patients with metastasized disease (*n* = 19), eight patients had a positive history of VTE.

We found significantly higher counts of activated platelet-derived (CD62P- and CD63– positive) microvesicles in soft tissue sarcoma patients with localized and metastasized disease with a positive history of VTE, compared to sarcoma patients without a history of VTE. Although soft tissue sarcoma patients with a history of VTE exhibited significantly higher platelet counts than patients without a history of VTE (Table 3), the overall amount of platelet-derived (CD61-positive) microvesicles was not significantly higher in patients with a positive history of VTE.

Since we demonstrated that there are no significant differences in CD62P- and CD63-positive microvesicles between the different soft tissue sarcoma grades (Fig. 2), we consider the significant differences between the groups with and without a history of VTE (Fig. 3) to be related to the patients’ positive history of VTE rather than to the grading of the tumor.

Furthermore, we showed that patients with a positive history of VTE have elevated numbers of activated platelet-derived (CD62P- and CD63-positive) microvesicles but similar overall counts of platelet-derived (CD61-positive) microvesicles. In agreement with these results, it has been proven that CD62P-positive microvesicles and CD63-positive microvesicles were elevated upon platelet activation by different agonists [7]. Interestingly, Villmow et al. found higher levels of CD62P-positive microvesicles in patients with myeloproliferative syndrome compared to controls, which might indicate that these microvesicles provide a catalytic surface for thrombin generation, thus explaining the observation that patients with myeloproliferative syndromes have an increased risk of arterial or venous thrombotic events. [28]. Furthermore, soluble P-selectin (CD62P) was found to be elevated in patients with hematological and breast cancer when compared to controls [29].

Interestingly, we did not find significant differences in TF-bearing (CD142-positive) microvesicles in patients with soft tissue sarcoma as compared to healthy controls. TF-bearing microvesicles have been found to be elevated in patients with colorectal cancer, but not in patients with breast cancer, which demonstrates that an elevation of TF-bearing microvesicles may not be a common finding in cancer patients [15, 30].

Furthermore, we did not detect significant differences in TF-bearing microvesicles in patients with and without VTE. Thus, we assume that the risk of VTE in patients with soft tissue sarcoma is not related to the release of TF-bearing microvesicles but rather to other factors such as the elevation of CD62P- and CD63-positive microvesicles (Fig. 3).

In our study, the collection of blood samples was carried out several months to years after the occurrence of thrombotic events, which indicates that the elevation of CD62P- and CD63-positive microvesicles does not result from but is possibly a reason for the occurrence of the VTE.

Finally, this study is to our knowledge the first to show that significantly more Annexin V-positive and endothelium-derived (CD62E-positive) microvesicles circulate in the peripheral blood of patients with G3 soft tissue sarcoma compared to patients with G2 soft tissue sarcoma. In addition, we found significantly higher values of activated platelet-derived (CD62P- and CD63-positive) microvesicles in sarcoma patients with a history of VTE, which might be a prognostic factor for the occurrence of VTE in soft tissue sarcoma patients.

The detection of these microvesicles might be an interesting new tool for early diagnosis of soft tissue sarcoma patients with increased risk for VTE, possibly facilitating VTE prevention by earlier use of thromboprophylaxis.

In this regard, Lee and Levine, analyzing the risks and outcomes of venous thromboembolism and cancer [31], argue that although patients with cancer and acute VTE who take vitamin K antagonists for an extended period are at increased risk of bleeding as well as of recurrent VTE, low molecular weight heparin (LMWH) may be a valid option reducing the risk of recurrent thromboembolism without increasing the risk of bleeding [32]. In contrast, Khorana et al., evaluating the benefit of outpatient thromboprophylaxis with LMWH in high-risk patients in a multicenter randomized study, recently showed that thromboprophylaxis with dalteparin in cancer patients is associated with a non-significantly reduced risk of VTE and significantly increased risk of clinically relevant bleeding [33]. Thus, although thromboprophylaxis is recommended for most patients hospitalized with active cancer, the use of outpatient thromboprophylaxis still remains controversial. Concerning this matter, Donnellan et al. argue that outpatient thromboprophylaxis may only be used in carefully selected high-risk ambulatory patients [34].

Thus, further studies need to be carried out assessing the prognostic value of high CD62P- and CD63-positive microvesicle counts with the occurrence of VTE as well as the outcomes of LMWH use for thromboprophylaxis in patients with soft tissue sarcoma.

## Conclusion

We found significantly higher levels of Annexin V-positive and endothelium-derived (CD62E-positive) microvesicles to be circulating in the peripheral blood of patients with G3 soft tissue sarcoma compared to patients with G2 soft tissue sarcoma. Furthermore, we showed that high counts of activated platelet-derived microvesicles correlate with the occurrence of VTE. Thus, the detection of these microvesicles might be an interesting new tool for early diagnosis of soft tissue sarcoma patients with increased risk for VTE, possibly facilitating VTE prevention by earlier use of thromboprophylaxis. However, further studies are required to assess the prognostic value and thrombogenicity of microvesicle levels in patients with soft tissue sarcoma.

## Additional file

Additional file 1: Representative fluorescence-activated cell sorting (FACS) dot plots. **A.** Forward and side scatter of isolated microvesicles stained with Fluorescein (FITC) Annexin V and Phycoerythrin (PE) anti-CD61 as well as TruCOUNT calibrating beads; MV = microvesicle gate. **B.** Events within MV-gate. Q1 = buffer / background. Q2 = Annexin V-positive and CD61-positive microvesicles. Q3 = Annexin V-positive microvesicles. (ZIP 121 kb)

## Abbreviations

BMI: Body mass index
CT: Computed tomography
FACS: Fluorescence-activated cell sorting
FITC: Fluorescein isothiocynate
Hb: Hemoglobin
LMWH: Low molecular weight heparin
MPNST: Malignant peripheral nerve sheath tumor
MRI: Magnetic resonance imaging
PE: Phycoerythrin
SD: Standard deviation
TF: Tissue factor
VTE: Venous thromboembolism
