# Supplementary figures and images for: Levels of activated platelet-derived microvesicles in patients with soft tissue sarcoma correlate with an increased risk of venous thromboembolism
Source: BMC Cancer. 2017 Aug 7;17:527. doi: 10.1186/s12885-017-3515-y (PMC5547532; doi:10.1186/s12885-017-3515-y)

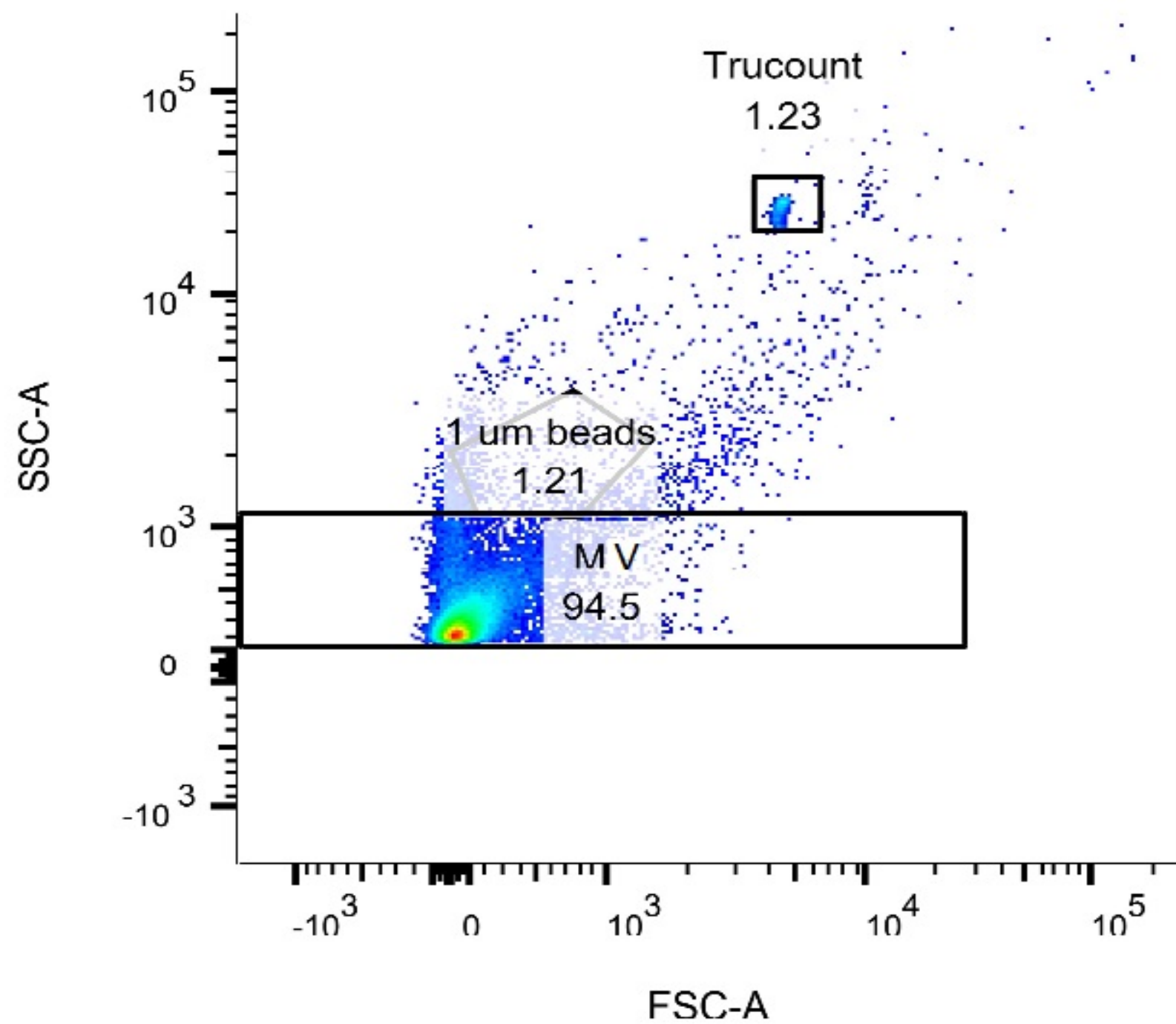

Supplement: Additional file 1: — Representative fluorescence-activated cell sorting (FACS) dot plots. A. Forward and side scatter of isolated microvesicles stained with Fluorescein (FITC) Annexin V and Phycoerythrin (PE) anti-CD61 as well as TruCOUNT calibrating beads; MV = microvesicle gate. B. Events within MV-gate. Q1 = buffer / background. Q2 = Annexin V-positive and CD61-positive microvesicles. Q3 = Annexin V-positive microvesicles. (ZIP 121 kb) [file 12885_2017_3515_MOESM1_ESM.zip › Additional file 1AR5.pdf]

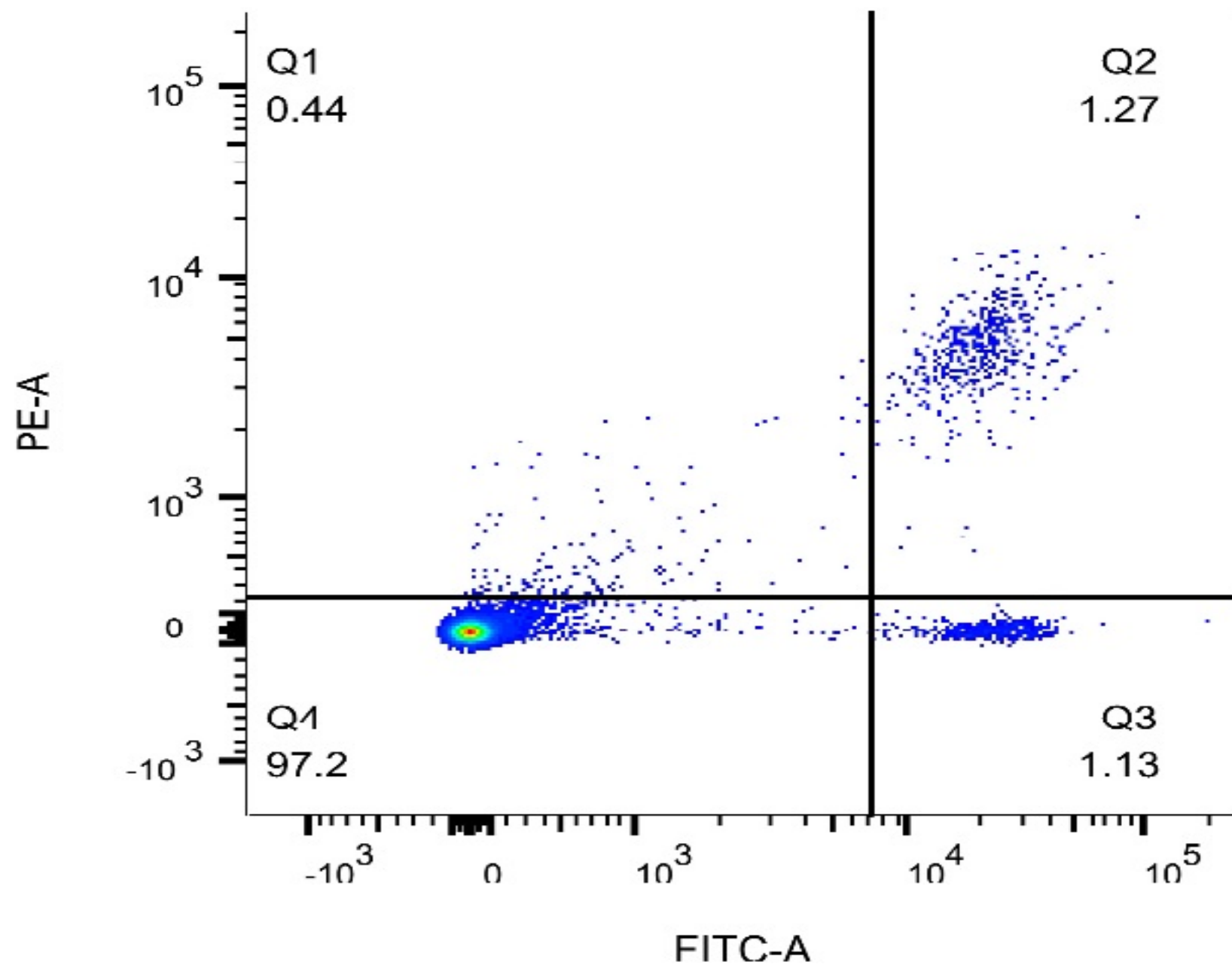

Supplement: Additional file 1: — Representative fluorescence-activated cell sorting (FACS) dot plots. A. Forward and side scatter of isolated microvesicles stained with Fluorescein (FITC) Annexin V and Phycoerythrin (PE) anti-CD61 as well as TruCOUNT calibrating beads; MV = microvesicle gate. B. Events within MV-gate. Q1 = buffer / background. Q2 = Annexin V-positive and CD61-positive microvesicles. Q3 = Annexin V-positive microvesicles. (ZIP 121 kb) [file 12885_2017_3515_MOESM1_ESM.zip › Additional file 1BR5.pdf]
